# A novel multi-layer perceptron model for assessing the diagnostic value of non-invasive imaging instruments for rosacea

**DOI:** 10.7717/peerj.13917

**Published:** 2022-08-17

**Authors:** Yingxue Huang, Jieyu He, Shuping Zhang, Yan Tang, Ben Wang, Dan Jian, Hongfu Xie, Ji Li, Feng Chen, Zhixiang Zhao

**Affiliations:** 1 Department of Dermatology, Xiangya Hospital of Central South University, Changsha, Hunan, China; 2 Hunan Key Laboratory of Aging Biology, Xiangya Hospital of Central South University, Changsha, Hunan, China; 3 National Clinical Research Center for Geriatric Disorders, Xiangya Hospital of Central South University, Changsha, Hunan, China; 4 School of Public Health, Kunming Medical University, Kuming, Yunnan, China; 5 Department of Biostatistics, School of Public Health, Nanjing Medical University, Nanjing, Jiangsu, China

**Keywords:** Rosacea, Reflectance confocal microscopy, VISIA, Dermoscopy, Multi-layerperceptron

## Abstract

**Background:**

Reflectance confocal microscopy (RCM), VISIA, and dermoscopy have emerged as promising tools for objective diagnosis and assessment of rosacea. However, little is known about the diagnostic value of these imaging systems for rosacea.

**Objectives:**

To assess the diagnostic value of RCM, VISIA, and dermoscopy for rosacea by establishing a novel multilayer perceptron (MLP) model.

**Methods:**

A total of 520 patients with rosacea and other facial diseases were included in this study. A total of 474 samples of dermoscopy data, 374 samples of RCM data, 434 samples of VISIA data, and 291 samples containing three data sources were collected. An MLP model was built with the total data to explore the association between the imageological features of each instrument and the probability of rosacea.

**Results:**

Our MLP model revealed that the area under the receiver operating characteristic curve (AUROC) values of RCM, VISIA and dermoscopy for diagnosing rosacea were 0.5233, 0.5646 and 0.7971, respectively. The integration of these three tools with clinical data could further improve the accuracy of the predictive diagnosis to 0.8385. For the imageological features of each tool, abnormalities (hyperkeratosis or parakeratosis) in the stratum corneum were effective variables for excluding rosacea (odds ratio [OR], 0.4333) under RCM. The indicators of rosacea under VISIA included overall severity of erythema, erythema involving the cheek or superciliary arch, visible red blood vessels, and papules (OR = 2.2745, 3.1592, 1.8365, 2.8647, and 1.4260, respectively). The candidate variables of dermoscopy included yellow background, white background, uniform distribution of vessels, branched vessels, and reticular blood vessels (OR = 0.4259, 0.4949, 2.2858, 3.7444, and 2.4576, respectively).

**Conclusions:**

RCM, dermoscopy, and VISIA each can present several imageological features and were of certain value for assisting rosacea diagnosis. The combined analysis of these three tools using our MLP model may be useful for improving the accuracy of diagnosing rosacea.

## Introduction

Rosacea is a chronic inflammatory skin disease characterized by various signs and symptoms, including facial flushing, erythema, papules, pustules, and phyma (Tan et al., 2017a). Some of these features, such as erythema and papules, can easily be confused with other facial dermatoses, such as lupus, eczema, and acne. The correct diagnosis of rosacea depends largely on the clinician’s subjective perception and experience (Tan et al., 2017b; Thiboutot et al., 2020). In recent years, the convolutional neural network has been utilized to objectively assess and classify rosacea based on the clinical photos of rosacea patients (Binol et al., 2020; Zhao et al., 2021). These networks are established based on a single type of image and require a huge amount of photos for training the network. In clinical setting, a variety of skin imaging instruments, such as reflectance confocal microscopy (RCM), dermoscopy, and VISIA have been utilized to assist in the diagnosis and measurement of rosacea (Logger et al., 2019). Dermoscopy is the most widely studied imaging instrument with the advantage of clearly displaying skin structures, particularly blood vessels for rosacea, from the surface to the mid-dermis (Logger et al., 2019; Micali et al., 2018). The most characteristic imaging feature of rosacea under dermoscopy is the presence of vessels arranged in a polygonal network (vascular polygons), which corresponds to superficial telangiectasis surrounding the follicles in histopathology (Lallas et al., 2014; Sgouros et al., 2018). Rosette signs and Demodex tails may also be observed under dermoscopy but are not specific for rosacea (Liebman et al., 2011; Rubegni et al., 2013; Segal et al., 2010). VISIA is another commercially available high-resolution facial imaging system that is particularly useful for showing the deep vascular component presenting as background erythema (Wang et al., 2018). This technique can also clearly display telangiectasis and has been applied for rosacea diagnosis, severity assessment, and therapy monitoring in previous studies (Micali et al., 2018; Schoelermann et al., 2016). RCM provides real-time microscopic images of the different skin layers deep in the papillary dermis (González et al., 2003). Existing studies describing the use of RCM in rosacea have mainly focused on the Demodex inhabitation of sebaceous follicles (Ruini et al., 2017; Sattler et al., 2015). However, Demodex inhabitation can also be observed in many other skin diseases (such as acne), and the clinical significance of Demodex in rosacea remains under debate (Falay Gur et al., 2018). Taken together, although these three non-invasive skin imaging tools have been used for the diagnosis of rosacea, comprehensive studies evaluating the diagnostic value of these techniques are lacking.

A multilayer perceptron (MLP) is a type of feedforward artificial neural network, which was generally used for complex issues. It consists of input, hidden and output layers. The MLP can discover complex nonlinear relationships between factors (input) and outcomes (output) (Plumb et al., 2005). In clinical applications, the MLP model has irreplaceable advantages in the analysis of multiple data sources. In clinics, collecting comprehensive clinical data and images for all patients are quite difficult, and traditional analysis might fill in the missing data, which often introduces uncertainty and makes the prediction unstable. By contrast, the MLP does not require to fill in the missing data because these missing parts would be automatically replaced by other information that is more reliable and has greater potential for clinical application. As a result, in the current work we built an MLP model to assess the reliability of these three types of non-invasive imaging tools for the diagnosis of rosacea.

## Materials & Methods

### Study design and population

We performed an observational, cross-sectional study among outpatients who visited the Department of Dermatology, Xiangya Hospital from May 2018 to December 2018. Patients with rosacea were included in the patient group, whereas those with other facial diseases characterized by facial erythema, papules, telangiectasis, such as acne, eczema, lupus, photoaging, and glucocorticosteroid-induced dermatitis were included in the control group. The study protocol was reviewed and approved by the ethics committee of Xiangya Hospital (IRB approval number 201404316). Written informed consent was obtained from all the patients.

### Data sources and processing

#### Clinical data

Rosacea was diagnosed based on the criteria of the National Rosacea Society Expert Committee in 2017. The diagnosis of rosacea and all other diseases was independently made by two dermatology experts in the clinic (based on clinical inspection only).

#### Instrumental evaluation

Digital images from the VISIA-CRTM system, RCM and X10 dermoscopy (Dermlite Hybrid^®^, 3 Gen, San Juan Capistrano, CA, USA) were collected. The central right malar region was chosen as the standard site for the RCM and dermoscopic examination. All these images were independently assessed and graded by two dermatologists. Dermoscopic images were evaluated using a set of three parameters (with a total of nine subitems) (Errichetti et al., 2020): (I) blood vessel morphology (dotted, curved, linear, branched, or reticular), blood vessel distribution (uniform or unspecific); (II) background (yellow, white, or reddish); and (III) follicular findings (yellowish halo, dilation of orifice, or pustules). A set of four parameters (with a total of ten subitems) was evaluated for RCM images and videos: (I) epidermis (stratum corneum abnormality, spongiform edema, and epidermal thickness); (II) interface change; (III) blood vessels (density, diameter, blood flow speed, and morphology); (IV) Demodex (percentage of affiliated follicles, and maximum number of Demodex mites in a single follicle). For VISIA examination, erythema-directed digital photography was equipped with the VISIA-CRTM system to enable the separation of the unique color signatures of red skin components (RBXTM system). The presence/degree of background erythema and the presence/location of telangiectasis and papules under the VISIA-CRTM system were recorded. All these imageological parameters and subitems were inputted into an MLP as candidate factors for further analysis.

### Statistical analysis

Continuous variables are summarized as mean and standard deviation (SD), and categorical variables are described as frequency (n), and proportion (%) and were evaluated using the Chi-square test. We did not perform missing data imputation. For the MLP, the missing value was labeled as zero and the other values were adjusted as value plus one.

#### Multilayer perceptron

Our MLP model was built with one hidden layer; the model structure and parameters are shown in Fig. S1. The optimizer was set as the RMSProp optimizer (Kurbiel & Khaleghian, 2017) with an learning rate of 0.001. A dropout and an early stopping strategy were utilized to prevent overfitting. Training was performed for up to 400 epochs, where the training loss reached a plateau. The area under the receiver operating characteristic curve (AUROC) and Kappa coefficient were used to adjust the class imbalance and assess the performance of the MLP model (Fatourechi et al., 2008). The data were randomly split into training and validation sets. The MLP model was trained in the training set and evaluated in the validation set. Model performance was assessed with different sources (demographic data, clinical inspection, dermoscopy, RCM, and VISIA) alone and combining all of them. This process was repeated 1000 times and the 95% confidence interval (CI) of the AUROC was calculated (as shown in Fig. S2).

The MLP was implemented using Python version 3.6.3 by ML library Scikit-Learn (Pedregosa et al., 2011) and Keras (https://keras.io/).

#### Logistic regression

Logistic regression (LR) is a well-known modeling method. The popularity of LR may be attributed to its interpretability. Data from different sources and LR models adjusted for age and sex were used to test the association between candidate factors and the probability of rosacea. Correction for multiple tests was performed using the Bonferroni method (Bland & Altman, 1995). LR analysis was performed using the R-language (version 4.0.1). *p* values less than 0.05 were considered statistically significant unless otherwise stated.

## Results

### Data analysis

#### Sampling

**Figure 1:**
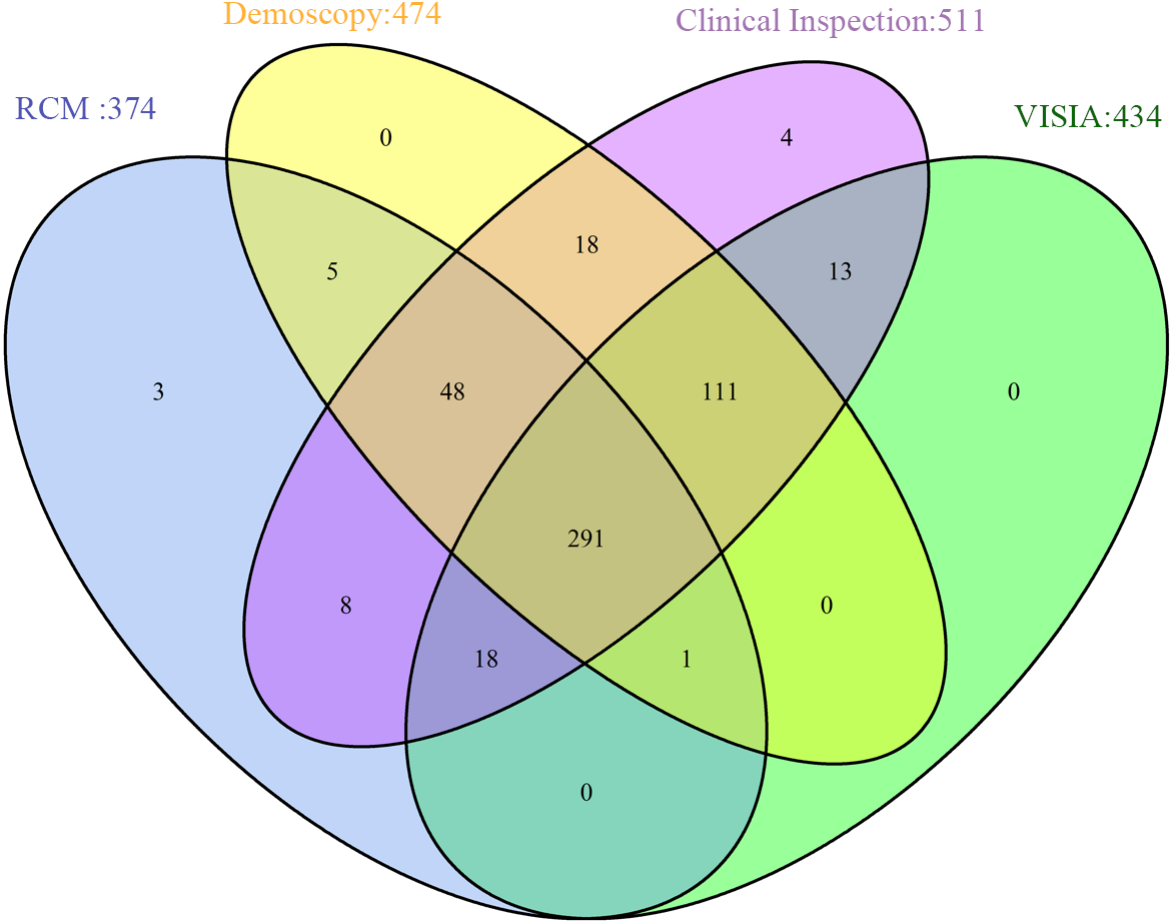
Data distribution from different sources.

Except for the random deletion of variables, the data contained block deletion are shown in Fig. 1. Among all the 520 patients, 474 underwent dermoscopic examination; 374, RCM data; 434, VISIA analysis, and 511, clinical inspection. A total of 291 patients underwent all these four examinations.

#### Demographic data analysis

As shown in Table 1, no statistically significant difference was found in age between the rosacea and control groups. However, a statistically significant difference was noted in the sex distribution (*p* = 0.00178).

**Table 1:** Demographic characteristics of patients and main imageological features of these three types of instruments in the rosacea and control groups.

Demographic characteristics	Rosacea	Control	*P*-value
Age	32.66 ± 11.54	31.5 ± 12.44	0.3247
Gender			
Female	333	119	0.00178
Male	37	31	

Imageological features		**Rosacea**		**Control**		***P*** **-value**
		N	%	N	%	
**Dermoscopy**						
Background	Yellow	125	33.8	51	49.5	0.003
	White	98	26.5	47	45.6	<0.001
	Reddish	130	47.8	19	33.9	0.058
Blood vessels	Uniform distribution	151	56.1	16	25	<0.001
	Dotted	27	10	17	25.8	0.001
	Curved	5	1.9	3	4.5	0.2
	Linear	75	27.9	25	37.9	0.112
	Branched	175	47.3	27	26	<0.001
	Reticular	178	48.1	29	27.9	<0.001
Follicular findings	Yellowish halo	117	41.8	21	26.9	0.017
	Dilation of orifice	17	6.1	4	5.1	0.754
	Pustules	46	16.4	10	12.8	0.438
**VISIA**						
Background erythema	Forehead	158	52.1	54	45.8	0.239
	Intercilium	235	77.6	68	57.6	<0.001
	Superciliary arch	228	75.2	58	49.2	<0.001
	Cheek	297	98	100	84.7	<0.001
	Perioral	142	46.9	47	39.8	0.192
	Chin	59	19.5	17	14.4	0.225
	Periorbital	265	87.5	85	72	<0.001
	Nose	240	79.2	79	66.9	0.008
	Preauricular	13	4.3	8	6.9	0.281
Overall erythema (Level 0–3)		2.426 ± 0.759		1.846 ± 1.039		<0.001
Papules and pustules (Level 0–3)		2.683 ± 1.190		2.214 ± 1.089		<0.001
Visible capillary		212	67.5	51	42.5	<0.001
**RCM**						
Stratum corneum abnormality		57	20.9	40	39.6	<0.001
Spongiform edema		220	80.6	86	85.1	0.48
Thickness of epidermis (*um*)		69.975 ± 15.555		67.413 ± 14.623		0.354
Interface change		22	8.1	11	11	0.381
>3 Demodex mites in single follicle (superficial)		56	20.8	19	19.4	0.764
>3 Demodex mites in single follicle (deep)		62	23.8	14	15.1	0.077
Density of vascular (Level 0–3)		1.425 ± 1.169		1.145 ± 1.216		0.084
Vasodilation (Level 0–3)		0.822 ± 1.087		0.678 ± 1.13		0.255
Blood flow speed (Level 0–4)		3.009 ± 1.073		2.767 ± 1.06		0.031
Vascular morphology	Branched	75	30	17	18.3	0.029
	Linear	216	86.4	79	84.9	0.73
	Curved	10	4	6	6.5	0.338
	Reticular	34	13.6	4	4.3	0.015

#### Imageological features of three types of instruments

The main imageological features of rosacea were identified and are listed in Table 1.

##### Dermoscopy.

Evenly distributed reticular blood vessels (vascular polygons) and a yellowish halo around the follicle were the most important imageological characteristics of rosacea under dermoscopy. The rosacea group exhibited significantly more branched and reticular blood vessels and fewer dotted capillaries than did the control group. A yellow and white background on the dermoscope can be helpful for excluding rosacea. Most cases of dermoscopy also exhibited pustules, which, however, did not show any significant difference between the rosacea and control groups.

##### RCM.

RCM examination of the rosacea revealed non-specific features. An important characteristic feature was the presence of capillaries and venules located in the dermis. However, neither the vascular density nor the vasodilation level was significantly different between the rosacea and control groups. The blood flow speed of patients with rosacea was significantly higher than that of controls, which might contribute to the background erythema in rosacea. Although likely incidental, reticular blood vessels (vascular polygons), as described on dermoscopy, were also observed under RCM (Video S1). Most RCM cases also exhibited telltale presence of Demodex mites within the follicular infundibulum. The percentage of hair follicles affected by three or more Demodex mites was higher in the rosacea group than in the control group; however this difference was not statistically significant.

##### VISIA.

**Figure 2:**
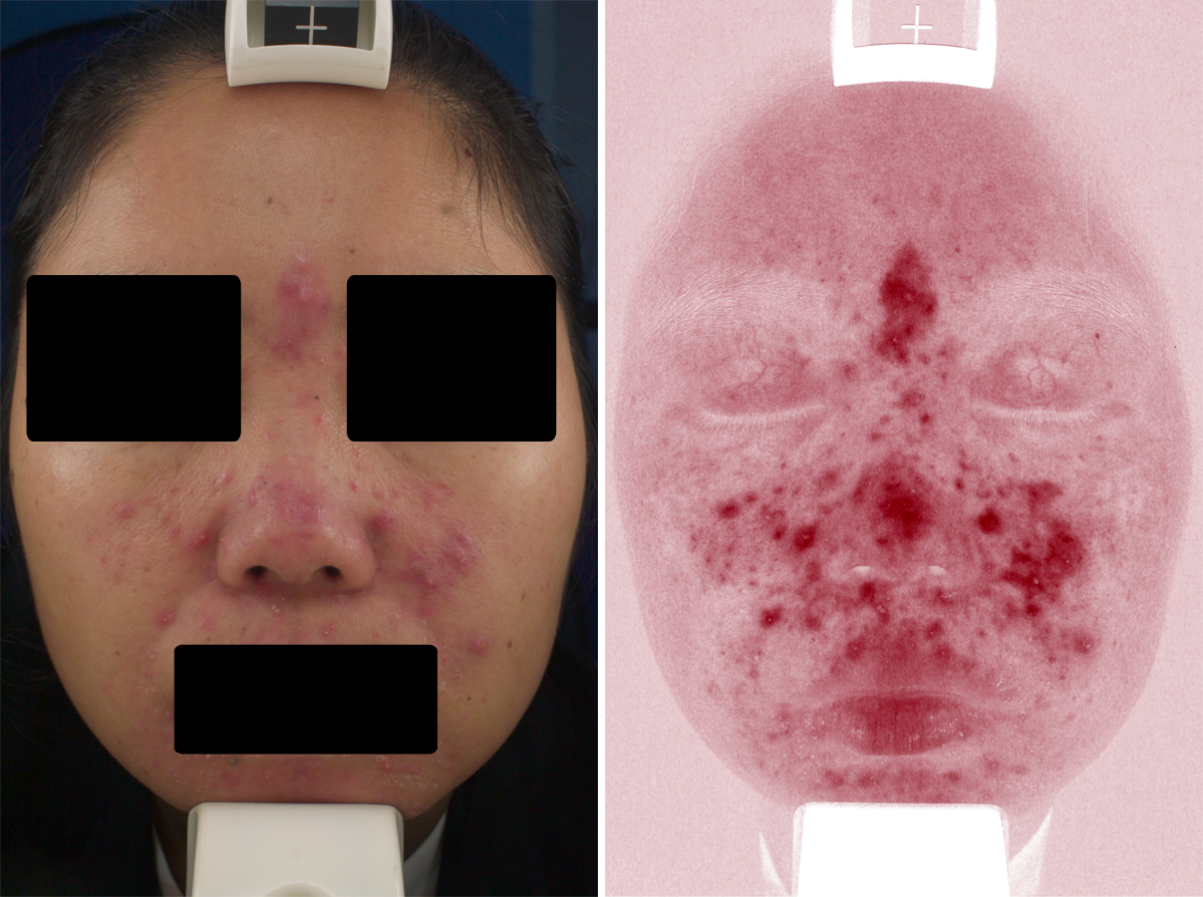
Representative VISIA images of rosacea patient.

Under the VISIA skin analysis system, rosacea was characterized by confluent and diffuse background erythema, telangiectasis, or a combination thereof. According to our results, telangiectasis was preferable to be located at the upper eyelid in rosacea, and the background erythema was more likely to implicate the superciliary arch, glabellum, periorbita, and nose. In addition, papulopustular lesions were detected using VISIA (Fig. 2).

### Process and results of multi-layer perception analysis

As shown in Table 2, the AUROC of demographic data, clinical inspection, RCM, VISIA, and dermoscopy were 0.5159 ± 0.0597, 0.6901 ± 0.0903, 0.5259 ± 0.0597, 0.4736 ± 0.0648, and 0.7573 ± 0.0505, respectively. Among these, dermoscopy achieved the highest AUROC. The integration of all the five different data sources achieved a prediction accuracy of 0.8385 ± 0.0436, which was much higher than each of them alone. The same results were also observed in the Kappa coefficient; a single source of demographic data, clinical inspection, RCM, and VISIA, which showed agreement between the true label and model prediction class by chance (Kappa coefficient = 0). The highest Kappa coefficients were 0.9258 ± 0.0536 in the training set and 0.5478 ± 0.0851 in the validation set, achieved by the integration of all five different data sources (Table 3).

### Univariate and multivariate LR analyses of the candidate factors for each imaging tool

To further screen for positive variables for each instrument, we performed univariate and multivariate LR analyses. After adjustment for demographic data, results showed positive findings in both the clinical inspection and the three imaging instruments (Fig. S2). The variable with the highest odds ratio value in the clinical inspection was persistent erythema on the convex areas of the face. An abnormality in the stratum corneum was the most effective variable under RCM and was a protective factor against rosacea. The positive variables for VISIA included the overall severity of erythema, involvement of the cheek and superciliary arch, and visible red blood vessels or papules, which were all high-risk variables for rosacea. The positive variables for dermoscopy included a yellow or white background, and a uniform distribution of vascular branched vessels and reticular blood vessels (vascular polygons). Among them, a yellow or white background was a protective variable for rosacea, whereas the aforementioned vascular morphologies were high-risk variables for rosacea (Table 4).

**Table 2:** The AUROC of demographic data, clinical inspection, RCM, VISIA and dermoscopy by multi-layer perception analysis.

**Source of characteristics**	**Training set**	**Validation set**
Demographic data only	0.5237 ± 0.0173	0.5159 ± 0.0597
Clinical inspection only	0.6965 ± 0.0725	0.6901 ± 0.0903
RCM only	0.5559 ± 0.0172	0.5259 ± 0.0597
VISIA only	0.5023 ± 0.369	0.4736 ± 0.0648
Dermoscopy only	0.7872 ± 0.0194	0.7573 ± 0.0505
Dermoscopy + clinical inspection	0.8621 ± 0.0206	0.8219 ± 0.0485
Dermoscopy + clinical inspection + RCM	0.9212 ± 0.0163	0.8191 ± 0.0464
Dermoscopy + clinical inspection + VISIA	0.9487 ± 0.0140	0.8407 ± 0.0449
Combine all sources	0.9964 ± 0.0058	0.8385 ± 0.0436

**Table 3:** The Kappa coefficient of demographic data, clinical inspection, RCM, VISIA and dermoscopy by multi-layer perception analysis.

**Source of characteristics**	**Training set**	**Validation set**
Demographic data only	0	0
Clinical inspection only	0.0032 ± 0.0251	0.0029 ± 0.0235
RCM only	0	0
VISIA only	0	0
Dermoscopy only	0.4125 ± 0.0552	0.3651 ± 0.1044
Dermoscopy + clinical inspection	0.4985 ± 0.0494	0.4385 ± 0.0969
Dermoscopy + clinical inspection + RCM	0.6864 ± 0.0384	0.5066 ± 0.0928
Dermoscopy + clinical inspection + VISIA	0.7475 ± 0.0531	0.5434 ± 0.0919
Combine all sources	0.9258 ± 0.0536	0.5478 ± 0.0851

**Table 4:** Logistics regression analyses of the candidate factors for each imaging tool.

	**Variables**	**Single factor**		**Multiple factor logistics**	
		**OR**	***P value***	**OR**	***P value***
Demographic data	Age	1.0086(0.9923,1.0256)	0.3084	–	–
	Gender	0.4265(0.2534,0.7212)	0.0014	-	–
Clinical inspection	Phymous changes	3.5691(2.1689,6.1395)	1.47E−06	3.4116(2.064,5.89)	8.10E−06
	Persistence erythema	22.5974(11.8157,47.1048)	4.95E−19	24.7898(12.7358,52.4947)	6.73E−19
RCM	Stratum corneum	0.4024(0.2455,0.6608)	0.0003	0.4333(0.2608,0.7218)	0.0012
VISIA	Overall erythema	2.0576(1.6166,2.6381)	7.14E−09	2.2745(1.7516,2.9848)	1.79E−08
	Cheek	3.0359(2.1294,4.3868)	1.56E−09	3.1592(2.1834,4.6476)	2.81E−08
	Visible capillaries	2.8120(1.8301,4.3492)	2.75E−06	2.8647(1.815,4.5546)	9.19E−05
	Papules	1.4101(1.1737,1.7023)	0.0002	1.4260(1.1835,1.7276)	0.0029
	Superciliary arch	1.8067(1.3186,2.5025)	0.0003	1.8365(1.3357,2.5535)	0.0030
Dermoscopy	Background (white)	0.4293(0.2733,0.6751)	0.0002	0.4259(0.2666,0.6801)	0.0062
	Background (yellow)	0.5202(0.3339,0.8099)	0.0038	0.4949(0.3117,0.7839)	0.0493
	Blood vessels (reticular)	2.3976(1.5061,3.9025)	0.0003	2.2858(1.4258,3.7427)	0.0136
	Blood vessels (uniform distribution)	3.8309(2.1166,7.2910)	1.80E−05	3.7444(2.0355,7.2068)	0.0007
	Blood vessels (branched)	2.5594(1.5957,4.2105)	0.0001	2.4576(1.5218,4.0671)	0.0058

## Discussion

The correct diagnosis of rosacea can sometimes be challenging in clinical settings. The most widely accepted diagnostic criteria was released in 2017 by the National Rosacea Society Expert Committee based on the clinical features of rosacea (Gallo et al., 2018). However, the accuracy of rosacea prediction based on clinical inspection alone was only 0.690 according to our MLP model. As a result, objective detection instruments are required to improve the diagnostic accuracy. Although few studies have focused on utilizing non-invasive imaging technologies for rosacea, they were designed mainly for research purposes (*e.g.*, follow-up of new treatments) rather than for clinical diagnosis (Bageorgou et al., 2019; Schoelermann et al., 2016; Sparavigna, Tenconi & De Ponti, 2014). Additionally, most methods can only measure one or a few parameters of the complex symptoms of rosacea. Therefore, a combination of several instruments is desirable. In this study, we developed a novel MLP model that could comprehensively assess and integrate the characteristics of RCM, VISIA, and dermoscopy, and further screened possible candidate variables of each instrument for the diagnosis of rosacea.

Many previous reports have described the morphology of rosacea using RCM, dermoscopy, and VISIA (Lallas et al., 2014; Liebman et al., 2011; Turgut Erdemir et al., 2014; Turgut Erdemir et al., 2017). However, the sample sizes were relatively small and the inter-study outcome variability was large. A possible explanation for these discrepancies is that the measurement sites were not consistent. Based on our results, RCM examination of rosacea revealed mostly non-specific features, except that the abnormal stratum corneum represented an exclusionary feature for rosacea, which might be because rosacea barely affects the stratum corneum. One advantage of RCM is its ability to assess the blood vessels live. Increased cutaneous blood flow was observed in rosacea by RCM, but neither the density nor the diameter of the blood vessels revealed any significant statistical difference. The characteristic reticular blood vessels that were often observed under dermoscopy could also be notably seen in RCM, which has not been previously reported (Video S1). According to our results, dermoscopy and RCM findings were in concordance with each other in many cases, which could explain why the integration of RCM into MLP the model did not significantly improve the AUROC of prediction. Another advantage of RCM is its ability to detect the *Demodex* (Harmelin et al., 2014). Many previous studies have focused on quantifying *Demodex* in each follicle and the percentage of follicles affected by *Demodex* (Casas et al., 2012; Turgut Erdemir et al., 2017). However, *Demodex* inhabitation is not specific to rosacea and the clinical significance of *Demodex* colonization in rosacea is still under debate (Falay Gur et al., 2018; Lacey et al., 2018). Consistent with previous studies, most rosacea cases in this study also exhibited the presence of *Demodex* within the follicular infundibulum, which showed no statistical difference compared with that of the control group.

According to previous studies, the reticular blood vessels (vascular polygons) are the most significant dermoscopic characteristics of rosacea (Lallas et al., 2014). An interesting novel finding under dermoscopy was that most rosacea cases exhibited pustules that might be invisible to the naked eye. Further studies with larger sample sizes are encouraged to confirm these findings.

Because deep localized blood vessels could also be involved in rosacea, the RCM and dermoscopy are not always effective. As a result, some previous studies have attempted to utilize VISIA for the analysis of the overall erythema of rosacea (Micali et al., 2018; Schoelermann et al., 2016). The advantage of VISIA over other instruments, such as RCM and dermoscopy, is that VISIA can provide a full view of the whole face instead of focusing on a specific area. We used the VISIA system to evaluate the deep blood vessels of rosacea by assessing the confluent and diffuse background erythema, and found that the severity of the background erythema and cheek involvement were supportive evidence for rosacea diagnosis. Telangiectasis is another important presentation of rosacea under VISIA, which can often be observed in control subjects with sun-damaged facial skin (such as photoaging) or glucocorticosteroid-induced dermatitis (Helfrich et al., 2015; Lahiri & Coondoo, 2016; Wang et al., 2019). However, the telangiectasis displayed by VISIA for rosacea were preferably located in the upper eyelid (Fig. 2). Moreover, based on our results, the blood vessels on the superciliary arch are an important feature of rosacea in VISIA.

Similar to other well-known artificial intelligence studies, the exact factors that play their roles in MLP remain elusive. How to open this “black box” was still one of the biggest challenges in the field of artificial intelligence. In this study, we used LR regression analysis, which partly explained the influence of factors on the model. However, the potential interactions between these factors and their nonlinear effects require further exploration. Moreover, owing to the limited sample size and the different sources that may have duplicate information, our model has a trend of overfitting for the integrated multi-source model. Further studies with larger or multicenter databases are encouraged to validate and modify the MLP model.

## Conclusion

RCM, dermoscopy, and VISIA can present several imageological features and were of certain value for rosacea diagnosis. The combined analysis of the three non-invasive imaging tools, namely dermoscopy, RCM, and VISIA, using our MLP model may be useful for improving the accuracy of diagnosing rosacea.

##  Supplemental Information

10.7717/peerj.13917/supp-1 Supplemental Information 1 The flow chart of multi-layer perception analysis the missing value was labeled as zero and other values changed as value plus one. (A) Data were randomly split as training set and validation set. (B) The MLP model was built in the training set. (C) Model performance was assessed with different sources only and combines all sources respectively. Repeat A to C 1,000 times.

10.7717/peerj.13917/supp-2 Supplemental Information 2 Univariate logistic regression and multivariate logistic regression adjusted with age and gender were performed with different sources of data

10.7717/peerj.13917/supp-3 Supplemental Information 3 A video showing reticular blood vessels around hair follicles of RCM

10.7717/peerj.13917/supp-4 Supplemental Information 4 Raw Data The demographic characteristics and main imageological features of the 3 types of instruments in rosacea and control group before statistical analysis.

10.7717/peerj.13917/supp-5 Supplemental Information 5 Python code for MLP

## Additional Information and Declarations

### Data Availability

The following information was supplied regarding data availability: The raw data are available in the Supplementary Files.
