# Supplementary figures and images for: A novel multi-layer perceptron model for assessing the diagnostic value of non-invasive imaging instruments for rosacea
Source: PeerJ. 2022 Aug 17;10:e13917. doi: 10.7717/peerj.13917 (PMC9392450; doi:10.7717/peerj.13917)

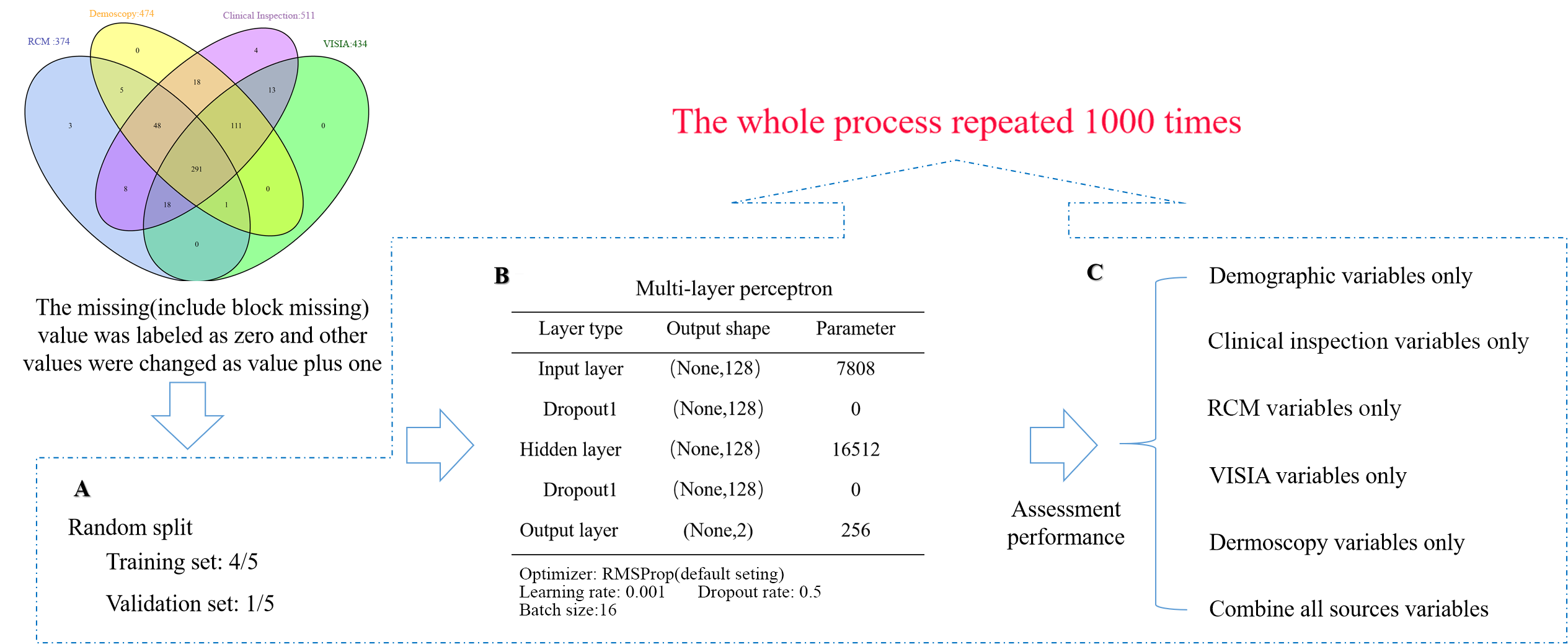

Supplement: Supplemental Information 1 — the missing value was labeled as zero and other values changed as value plus one. (A) Data were randomly split as training set and validation set. (B) The MLP model was built in the training set. (C) Model performance was assessed with different sources only and combines all sources respectively. Repeat A to C 1,000 times. [file peerj-10-13917-s001.png]

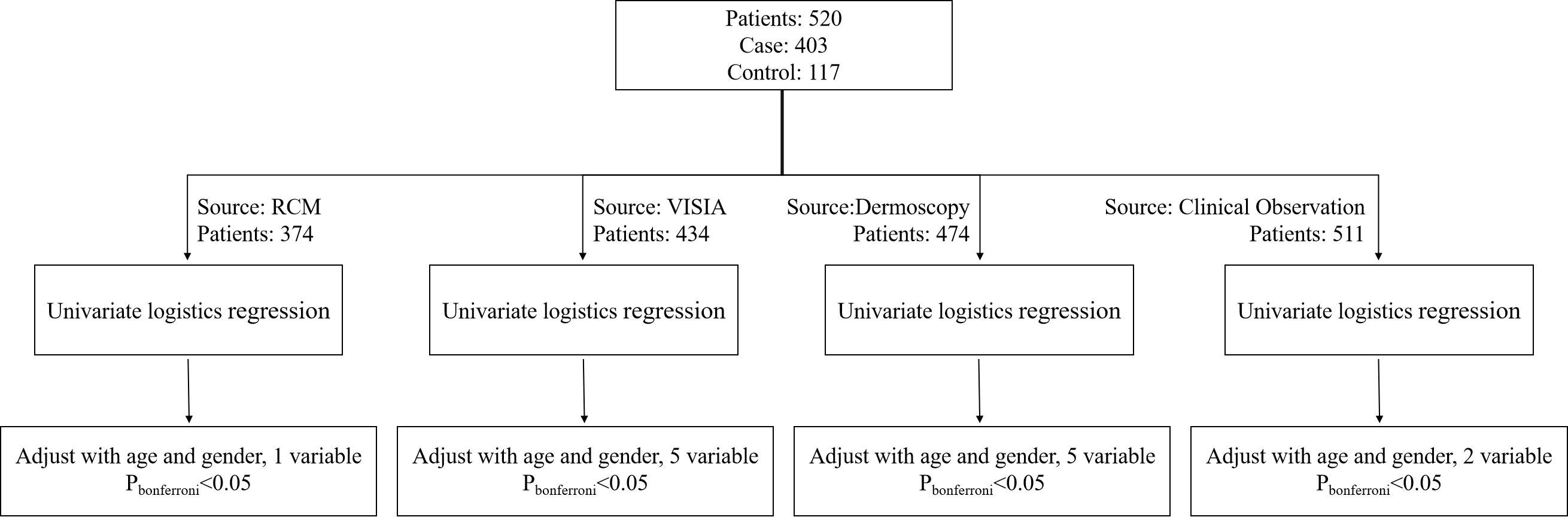

Supplement: Supplemental Information 2 [file peerj-10-13917-s002.png]
